# Donor conception, direct-to-consumer genetic testing, choices, and procedural justice: an argument for reform of the Human Fertilisation and Embryology Act 1990

**DOI:** 10.1093/medlaw/fwae028

**Published:** 2024-07-27

**Authors:** Caroline A B Redhead, Lucy Frith

**Affiliations:** Centre for Social Ethics and Policy, Law Department, The University of Manchester, UK; Centre for Social Ethics and Policy, Law Department, The University of Manchester, UK

**Keywords:** direct-to-consumer genetic testing, donor anonymity, donor conception, law reform, procedural justice, retrospective law reform

## Abstract

In this article, using theories of procedural justice and ‘slow violence’, we consider potential reform of the Human Fertilisation and Embryology Act 1990. Our theoretical discussion is underpinned by findings from the ConnecteDNA project, exploring how people affected by donor conception experience direct-to-consumer genetic testing (DTCGT). The negative impacts of DTCGT, especially shock discoveries about the circumstances of someone’s conception in adulthood, are linked to donor anonymity, and how its continued protection is experienced as a barrier to the rights and agency of donor-conceived people. We focus on two key issues relating to the donor information access process set out in section 31ZA of the 1990 Act. The first is that it excludes certain cohorts of donor-conceived people, creating inequalities of access to donor information. The second is the impact of the use of DTCGT to search for that information. We discuss what a procedurally just process of law reform would look like, concluding that, whatever (prospective) approach to donor anonymity is taken, the donor information access process *should be the same for all donor-conceived people*. We thus argue that, even were the *status quo* to be maintained, reform of the donor information access process with retrospective effect would be required.

## I. INTRODUCTION

Legislation to regulate assisted conception was first discussed in England and Wales in the early 1980s. At that time, it was considered that gamete donor anonymity would protect donors and recipient couples from legal complications and from the emotional difficulties of ‘introducing a third party into what ought to be an exclusive relationship’ (noting that at that time it was predominately heterosexual couples who had access to medically assisted reproduction services).^1^ Over four decades later, legal approaches and cultural values have changed dramatically. However, elements of gamete donor anonymity (such as prohibiting access to information during childhood) remain a feature of the regulation of donor conception in the UK, as well as in many other jurisdictions.

Technological capability has also changed during that time. In particular, the existence and relative affordability of direct-to-consumer genetic testing (DTCGT) now offers donors and the donor-conceived community the means to circumvent the legal frameworks that guarantee anonymity. Our qualitative study, the ConnecteDNA research project, has explored how people affected by donor conception understand and experience the implications of DTCGT.^2^ Our findings show that DTCGT has changed how knowledge about donor conception is disseminated—who knows and how they know. Donor-conceived people have discovered the method of their conception through DTCGT, and its use has enabled people who were unknown to each other, but connected through donor conception, to identify and sometimes contact one another, often through a third person (such as the spouse, partner, or child of an historic sperm donor). The discovery of unexpected genetic connections often comes at an emotional cost and, for some, can be associated with psychological distress and disruption to family relationships.

Thus, for many people affected by donor conception, DTCGT offers new choices in relation to donor information. Participants in the ConnecteDNA project, however, offered varied, and often starkly opposing, views about their merits and disadvantages. Parents of donor-conceived children wondered, for instance, whether they should use DTCGT to test their young child (perhaps too young to give meaningful consent) or wait and support them to use DTCGT only if, or when, the child expressed an interest in doing so. Or whether, instead, they should abide by the official age restriction (which, in the UK, is 18 years) to access identifying information?^3^ If they were to opt for early use, their child might discover, and be able to grow up with, knowledge of, and, possibly, connections to, donor relatives.^4^ For parents opting to wait, however, there were differences of opinion about the level of understanding their child should have about the potential challenges involved in using DTCGT. These might include the implications of online data sharing, and the lack of any counselling or psychological support (unless those who need support are able to arrange and pay for it themselves). In contrast, those applying for information through the Human Fertilisation and Embryology Authority (HFEA) are entitled to ‘a suitable opportunity to receive proper counselling about the implications of compliance with the request’.^5^ Hence, the choices offered by DTCGT are sometimes experienced as moral dilemmas with some choices perceived as having potentially harmful consequences.

In this article, using data from the ConnecteDNA project, we consider how DTCGT is used and experienced by donors, donor-conceived people and parents of donor-conceived people.^6^ Recognizing that the use of DTCGT services comes with the potential for disruptive, sometimes psychologically harmful, consequences for those affected by donor conception, we build a picture of the tensions that currently exist between laws protecting donor anonymity and the interests of donor-conceived people. These tensions are experienced both by donor-conceived people who have no legal route to access information about their genetic origins, and by those who feel that the age-related thresholds delaying access to a donor’s information until a donor-conceived person is 18 years old operate against their children’s best interests. Building our argument on the experiences of those affected by donor conception, historically marginalized in the regulation of assisted conception, we turn to theories of procedural justice to explore their sense that they are unfairly treated by the law regulating donor conception, the Human Fertilisation and Embryology Act 1990 (as amended) (the 1990 Act).

Procedural justice is a notion typically associated with the legitimacy of criminal justice authorities and the fairness of criminal justice processes, and these areas are the focus of much of the procedural justice literature. However, the concept of procedural justice is neither limited to the doctrinal analysis of processes and procedures, nor to the criminal justice arena. Anthony Bottoms and Justice Tankebe in their recent work, for instance, describe procedural justice as, ‘an everyday phenomenon, of which we all have experience’, and note that procedural justice (or injustice) is delivered in a wide variety of social contexts.^7^ Our interest here is in using theories of procedural justice to interrogate and evaluate, from a normative perspective, the regulatory process governing access by donor-conceived people in the UK to information about their donor. This process is set out in section 31ZA of the 1990 Act. Using qualitative data from the ConnecteDNA research to inform our discussion, we argue that the rules set out in section 31ZA should be reformed and that the law reform process should not preclude consideration of the retrospective removal of donor anonymity.

Drawing on recent procedural justice literature, we outline two related arguments. First, we suggest that the continued (albeit partial) legal protection of gamete donor anonymity in the UK diverges from now widely accepted normative standards. It is clear that, for many decades, legislators have been aware of, and, in some circumstances (adoption being one) attentive to, the importance to an individual of knowing their genetic origins.^8^ We also note the potentially harmful consequences of the use of DTCGT, which, for many donor-conceived people, represents the only means of finding and accessing the information they seek. Secondly, we suggest that serious consideration must be given to the removal of donor anonymity with retrospective effect. We argue that there is no other way in which to recognize the importance to *all* donor-conceived people of information about their donor. To inform the law reform process, we call for a participatory, evidence-based review of the regulation of donor information provision to donor-conceived individuals. Our contention is that, in the very specific circumstances we present in our discussion, law reform with retrospective effect offers a mechanism by which a more equitably balanced system of choices for donor-conceived people (and their parents) might be achieved. A crucial part of this process would be to weigh the relative rights and interests of historic donors who, at the time of their donation, were assured that their identity would not be disclosed.

The article proceeds as follows. In Section II, we describe how the regulatory landscape in England and Wales approaches the issue of donor anonymity, looking briefly at the historical reasons for this. We then introduce the ConnecteDNA project, which examines the use of DTCGT by, and its impact on, gamete (egg, sperm, and embryo) donor-conceived adults, donors, and parents of donor-conceived people.^9^ Our research focuses particularly on the impact of DTCGT on donor anonymity, which continues (theoretically, at least), to be maintained until donor-conceived people reach the age of 18 years.^10^

We move, in Section III, to explore theories of procedural justice. Drawing on the philosophical literature, we briefly outline the theoretical background, within which we then situate and assess the process (described in section 31ZA of the 1990 Act) governing access by donor-conceived people to information about their donor (the donor information access process). We contend that, in denying some donor-conceived people access to information about their donor based on the date of the donation from which they were conceived, the donor information access process fails both to uphold their rights and to respect their dignity. For this reason, we suggest the donor information access process is procedurally unjust. To develop and substantiate our argument, we turn to Rob Nixon’s theory of ‘slow violence’,^11^ and the work of Ashley Barnwell in applying Nixon’s theory to family secrets.^12^ Writing about environmental pollution, Nixon describes ‘a violence that occurs gradually and out of sight, a violence of delayed destruction that is dispersed across time and space, an attritional violence that is typically not construed as violence at all’.^13^ He characterizes it as ‘slow violence’, and calls for scholars to attend to the ‘uneventful injustices that slip beneath the radar, dismissed or postponed’.^14^ Barnwell argues that the notion of ‘slow violence’ is also relevant to the temporal dimensions of other sociological problems,^15^ and we consider it in the context of DTCGT’s disruption of legally protected gamete donor anonymity. We will also consider the parallels (and differences) between adoption and donor anonymity, in terms of state-sponsored secrecy.

Moving next to the options for reform of the 1990 Act, which are currently being considered, we describe and discuss, in Section IV, the HFEA’s recent consultation and proposals for law reform.^16^ The Assisted Reproductive Treatment Amendment Act 2016 in Victoria, Australia, which removed donor anonymity with retrospective effect, is referenced in the HFEA’s discussion documents,^17^ but the HFEA’s recommendations appear to rule out retrospective reform of the 1990 Act. Drawing again on theories of procedural justice, we suggest, in Section V, that it would be a mistake not to include, as part of a detailed review of the donor information access process, an analysis of the potential benefits and harms of removing donor anonymity with retrospective effect in the UK. We propose five principles to underpin this process. In Section VI, we conclude that, whatever the conclusion about removing donor anonymity, all donor-conceived people should be treated equally.

This article draws on literature and legislation that will be well-known to academics working in the field of donor conception. Our contribution is to present the hitherto unheard voices of those affected by donor conception, to bring theories of procedural justice and slow violence to bear in our discussion of the donor information access process and the proposed reform of the 1990 Act, and to develop an argument for the retrospective removal of donor anonymity based on this analysis.

## II. REGULATING DONOR ANONYMITY

### A. Legislating for donor anonymity: the Warnock Report

The drafting of the 1990 Act, which regulates assisted conception in the UK, was preceded by the work of a Committee of Inquiry into Human Fertilisation and Embryology, chaired by Dame Mary Warnock (the Warnock Committee), whose terms of reference included consideration of the social, ethical, and legal implications of developments in human fertilization and embryology.^18^

Members of the Warnock Committee ‘tried…to give due consideration both to public and to private morality’.^19^ They concluded that gamete donor anonymity ‘protects all parties not only from legal complications but also from emotional difficulties’, recommending that, as a matter of good practice, ‘any third party donating gametes for infertility treatment should be unknown to the couple before, during and after the treatment, and equally the third party should not know the identity of the couple being helped’.^20^ One of our participants, Patricia, who underwent fertility treatment at that time, felt, ‘the whole process was so new, I don’t think there was any understanding of it’ but has now, ‘as a much older woman, a huge understanding and awareness of all those implications’, which she describes as ‘a spider’s web’. She describes how she, ‘wrote to Dame Warnock, on my typewriter’ to express her strongly held view against donor anonymity: there ‘really aren’t many words that you can, you can use to describe how wrong it is … that nobody has the right to create a treatment to bring a child into the world where they can’t know whose eyes they’ve got, or whose hair they have, what illnesses they might be carrying’.

The Committee did acknowledge that:

[t]he sense that a secret exists may undermine the whole network of family relationships. [Donor-conceived] children may feel obscurely that they are being deceived by their parents, that they are in some way different from their peers, and that the men whom they regard as their fathers are not their real fathers. We have little evidence on which to judge this. But it would seem probable that the impact on children of learning by accident that they were born as a result of [donor conception] would be harmful-just as it would be if they learned by accident that they were adopted or illegitimate.^21^

However, despite also suggesting that ‘it is wrong to deceive children about their origins’, it sought a middle ground. This was to recommend that ‘the absolute anonymity of the donor’ should be maintained but legislation should provide that, on reaching the age of eighteen, the child should have access to basic (non-identifying) information about the donor, for example, ethnic origin and genetic health. For Patricia, ‘many parts of the HFEA’s policy [at that time] that felt wrong’, but ‘the biggest one [was] the fact that, however honest I was with my children, I couldn’t give them [the identifying information about their genetic father that] I considered to be the birthright of everybody’.

### B. The 1990 Act and beyond

Based on the recommendations of the Warnock Committee, the 1990 Act was drafted to ensure the anonymity of gamete donors, although the HFEA has always been obliged to keep a register of donors’ identifiable details.^22^ This remained the position until, in 2005, The Human Fertilisation and Embryology Authority (Disclosure of Donor Information) Regulations 2004 (2004 Regulations) came into force.

The 2004 Regulations required the HFEA, in certain circumstances, to disclose certain information about their gamete donor to applicants on request.^23^ There were a number of factors leading to this change. In 2002, in the case of *Rose v Secretary of State for health and Human Fertilisation and Embryology Authority*,^24^ (*Rose*) it was acknowledged that Article 8 of the European Convention for the Protection of Human Rights and Fundamental Freedoms 1950 (Article 8)^25^ was engaged with regard to identifying and non-identifying information about a biological parent. As a result, it was established that everyone should be able to establish details of their identity as a human being. Concurrently with *Rose*, a public consultation on the provision of information to donor-conceived people took place. In expressing support for the removal of anonymity for donor-conceived people, respondents expressed their view that information about someone’s origins is a basic human right, and important for the emotional needs of donor-conceived children.^26^ Our data support this. For example, Nia felt, ‘everyone has a right to know, biologically and DNA-wise, where they’ve started’, and Patricia described anonymity as, ‘wrong, plain and simple’.

The case of *Odièvre v France*,^27^ was also influential. In this case, the European Court of Human Rights confirmed that ‘people have a right to know their origins , […] derived from a wide interpretation of the scope of the notion of private life’ enshrined in Article 8.^28^ The 2004 Regulations, recognizing this right, enabled donor-conceived people conceived from donations made after 31 March 2005, from 18 years of age, to apply to the HFEA for their donor’s name, date and place of birth, appearance, and last known postal address.^29^ Beth, a donor-conceived participant in our study, felt that this was ‘a step in the right direction’, but that ‘18 is a really abstract age and that’s going to feel like a lifetime to a teenager growing up and literally trudging towards that arbitrary line’. The first cohort of identity-release donor-conceived people reached that line and were able to apply for this information in 2023 (assuming, of course, that they were aware of the circumstances of their conception and of their right to access it).^30^ Those born from donations made prior to 31 March 2005 have no legal route to access identifying information about their donor.

For those who are aware they were donor-conceived, the existence of DTCGT offers choices. Those born from donations made before 31 March 2005 can choose to use DTCGT, in conjunction with information shared on social media sites, to search for information about their donor and others to whom they are related through their donor (donor relatives). For example, Mark told us, ‘as soon as I found out [I was donor-conceived], within a week I think I’d ordered a DNA kit from Ancestry, and I sent that off … yes I did it straight away. I wanted to find out as much as possible as quickly as I could’. For post-2005 donor-conceived and their parents, DTCGT means they can search for their donor and other genetic relatives before they turn 18 years old and, thus, provides the opportunity to circumvent the regulatory framework protecting the donor’s anonymity during the childhood of the donor-conceived person.

The HFEA has acknowledged the disruptive effects of DTCGT. Specifically, in its 2023 consultation document, the HFEA recognized that,

the issue of accessing donor information and identifying donors, has become more urgent with the growing popularity of easily accessible, relatively affordable direct-to-consumer DNA testing and matching services which have revolutionised our ability to find our genetic relatives. Mainstream media and social media have shone a light on how these services can provide information to those who previously had no way of finding out their full genetic origins.^31^

Findings of the ConnecteDNA project inform an understanding of the use and experiences of DTCGT by donors and the donor-conceived community, particularly in the context of searching for information about donors and donor relatives. In so doing, the findings shed light on the failings of the donor information access process and support an argument for reform of the 1990 Act.

### C. Direct-to-consumer genetic testing and the ConnecteDNA project

DTCGT platforms, such as Ancestry and 23andMe,^32^ offer consumers a plethora of options^33^ in return for a fee and a sample of their DNA, usually provided by sending a saliva sample for analysis. Among the services available, are ‘relative finder’ and ‘matching’ services, which facilitate the building of family trees and connections with genetic relatives. DTCGT services are marketed as fun products, with a notable increase in advertising at certain times of the year, such as Christmas and Mother’s Day.^34^ The ConnecteDNA research shows that the rise in the use of DTCGT is affecting how information about donor conception is managed. In particular, we have found that DTCGT shifts patterns of knowledge about donor conception; increases flexibility regarding the age at which information about donor relatives is accessible; can lead to a growing role for non-professionals, including wider family members, in gatekeeping information about the circumstances of *others*’ conception; accentuates the impact of donor conception for donors’ and donor-conceived people’s relatives; and shapes, and is shaped by, the donor information access process.^35^

We carried out in-depth interviews with donors, donor-conceived people, and parents by donor conception, exploring participants’ experiences of donor conception and DTCGT.^36^ Participants were asked about the framework for regulating donor anonymity, the role of law in regulating DTCGT, and the terms and conditions, particularly with regard to privacy, that underpin DTCGT services. We also held stakeholder workshops,^37^ attended by donors, donor-conceived people, parents of donor-conceived people, and professionals working in the field of donor conception.^38^ In the workshops, options for reform of the 1990 Act were discussed, in line with the HFEA Legislative Reform Advisory Group’s discussion document.^39^ We also interviewed regulatory experts in the Netherlands and the Australian State of Victoria.

Our findings confirm that some of the more negative impacts of DTCGT in relation to donor conception, particularly shock discoveries in adulthood about the circumstances of someone’s conception, are directly connected to the practices of secrecy in relation to infertility, gamete donation, and donor conception. The influence of the Warnock Committee’s support for secrecy and its recommendation that the law should protect donor anonymity continue, by virtue of the donor information access process, to impact the lives of the donor-conceived community today.^40^ While our data show that views and experiences are complex and varied, it is clear that, for many, the donor information access process is experienced as a barrier to their (or their children’s) rights, their agency, and to the information they feel is constitutive of their identity.

## III. PROCEDURAL JUSTICE AND THE DONOR INFORMATION ACCESS PROVISIONS

As described above, our focus in this article is on section 31ZA of the 1990 Act, which we have described as the donor information access process. It is a statutory process governing access by a particular class of people to information (both identifying and non-identifying) about their biological parent(s). The information is held by the state (in the form of the HFEA), which proscribes access to it until the person to whose parent it relates is either 16 years, for non-identifying information, or 18 years, for identifying information. It is the identifying information which is the focus of our interest here. In using theories of procedural justice to examine the donor information access process, we examine discussions about procedures in legal philosophy, focusing on the important role that fair legal processes play in justifying the exercise of legal power.

### A. Theories of procedural justice: a summary

We understand a theory of procedural justice to be a normative theory which seeks to explain the moral basis for imposing procedural obligations on decision-makers (such as to collect, keep secure and, ultimately disclose information about gamete donors to those born of their donation) and the principles informing process design.^41^ Our purpose, in appealing to procedural justice, is to consider whether there is a continued normative basis for the protection of donor anonymity and, in so doing, both to evaluate the fairness of the donor information access process and to consider options for reform.

Theories of procedural justice in the law can broadly be categorized as utilitarian, outcome-based, or dignitarian.^42^ Each takes a different approach to the value of legal processes and procedures, some ascribing value only to the instrumental aspects (eg, achieving a legally correct outcome), and others assuming that there is broader, non-instrumental value in a fair process (such as ensuring that those with an interest in its outcome have a chance to participate in the decision-making). Ronald Dworkin, defending an outcome-based approach, argued that a utilitarian theory is unable to recognize the distinct kind of injury done when legal rights are not upheld, even where a procedural error is not deliberate. He offers the example of an innocent person, wrongly imprisoned for a crime, who suffers both the harm of imprisonment and the ‘moral harm’ of being denied their rights.^43^

While Dworkin does not suggest that avoidance of moral harm should be the priority in designing fair processes, he contends that an important function of processes (and, by implication, process design) is to confer protection against moral harm, understood as a procedural right.^44^ Dennis Galligan describes a ‘close and interesting relationship between rights and procedures’, arguing that a right is taken seriously only if there are procedures for its protection, although acknowledging that procedures which allow the risk of a less-than-perfect outcome are not thereby necessarily unjust, rather that it is a matter of balance and proportionality.^45^ Processes and procedures can therefore be understood to have non-instrumental value in and of themselves, independently of their outcome.^46^ Such dignitarian accounts of procedural justice are underpinned by Kantian ideas of rationality and responsibility, including that a human being should be accorded dignity and autonomy in the relationship between citizen and state, and regarded as a person, rather than an object about which decisions can be made.^47^ In the criminal justice context, for example, a procedurally just process would allow the defendant a voice even where their guilt were beyond doubt.^48^

Meyerson and MacKenzie have recently offered a new perspective on procedural justice.^49^ In constructing their account, they draw on relational theory, which understands humans as social creatures whose self-identities and sense of self-respect are constituted by, and enmeshed in, relationships with other individuals, groups, and institutions.^50^ Discussing relational theory in ethics and political philosophy, and relational accounts of procedural justice in social psychology and criminology, their account of procedural justice upholds the normative primacy of individuals but rejects social atomism.^51^ Thus, the rights, welfare, dignity, and autonomy of individuals matter, and impose constraints on the claims of social collectives. Meyerson and MacKenzie argue that relational theory draws specific attention to the moral significance of social oppression, marginalization, and inequalities of social power and standing, and their effects on individuals’ self‐identities, autonomy, and sense of self‐respect. They contend that legal processes matter to people by virtue of their capacity to enhance both the quality of their interpersonal interactions with authorities, independently of the outcomes to which they lead, and to enhance the quality of interpersonal interactions between individuals and groups.^52^

While they agree with the dignitarian focus on respect for the people within legal processes, Meyerson and MacKenzie reject the notion of purely rational agency. Building on feminist critique of the individualistic and rationalistic focus of much contemporary ethics and philosophy,^53^ they propose, instead, ‘an expanded conception of agency as social, embodied, and emotional as well as rational’.^54^ Further, and importantly for our purposes, they highlight ‘the critical role of social relationships in fostering respect and self-respect’, noting how these values can be undermined and eroded by social injustice, oppression, and inequality.^55^ In the context of relationships with authorities, their account of relational procedural justice requires that people be treated fairly and even-handedly, and with respect and dignity.^56^ Of particular importance is participation in decision-making processes, including being enabled to voice a point of view. These things are required to validate people’s sense of self-worth, to promote self-respect and a sense of identity.^57^ Meyerson and MacKenzie’s account of procedural justice thus reflects the broader theoretical understanding of people as fundamentally social creatures, whose sense of identity and of self (including feelings of self-respect and self-worth) are bound up with, and grounded in, their interpersonal relationships.^58^ Relational dynamics thus become a crucial aspect of legal processes and, even where resources are limited, participation is seen as ‘a fundamental requirement that cannot simply be balanced away in the service of timeliness and efficiency’.^59^

Kirsten Rundle similarly characterizes legal procedures, processes, and institutional forms as the ‘primary vehicle for relational contact between legal officials and legal subjects’ and, thus, as fundamental in terms of demonstrating a legally constituted entity’s commitment to the exercise of lawful authority rather than ‘mere power’.^60^ She emphasizes the reciprocity between government and citizen that this implies, the mutual ‘fidelity to law’ that must be achieved for legal institutions and actors (such as the HFEA, in the context of donor conception) to govern with authority rather than power, and to *engage* with legal subjects through the medium of rules, rather than simply acting on them.^61^ She suggests that doing the latter would not only convey indifference to legal subjects’ agency and rights to self-determination, but also ‘negate the reciprocal dynamics upon which the achievement of legal order itself depends’.^62^ Thus, in the relational account of procedural justice, a dynamic relationship between legal subject and legal authority is fundamental to the maintenance of lawful authority. Rundle contends that these relational demands are critical across what she describes as ‘the modalities of contemporary government’, by which she means not only rules-based processes, but also ‘any exercise of official power apt to affect an individual’s interests, broadly understood.^63^

There is a sense in our data that this relational dynamic is lacking in the context of the donor information access provisions, which act on both donor-conceived people (‘I think it’s very cruel to deny people agency when they’re forming their own identity. It’s very cruel indeed.’ Ed, donor) and on donors (‘Do [anonymous] donors want to be contacted by their children? Probably not. I just don’t really think the HFEA’s kind of accepted the reality [of DTCGT] and kind of taken a proactive approach to what is the right thing to do.’ Gareth, donor-conceived person). We inform our discussion about the donor information access process below by reference to the relational approach to procedural justice developed by Meyerson and MacKenzie.

### B. The donor information access process and procedural justice

The donor information access provisions, as discussed above, set out a process for donor-conceived people to apply for non-identifying information about their donor at the age of 16 years and identifying information at the age of 18 years.^64^ The focus of our analysis is section 31ZA(5), which provides that:

Regulations cannot require the Authority to give any information as to the identity of a person whose gametes have been used or from whom an embryo has been taken if a [clinic] was provided with the information at a time when the [HFEA] could not have been required to give information of the kind in question.

There are, consequently, three cohorts of donor-conceived people affected by the donor information access process: (i) those born of donations made on or after 1 April 2005 who can access identifying information about their donor at the age of 18 years; (ii) those born of anonymous donations made between 1 August 1991 and 31 March 2005, whose information is stored by the HFEA but to whom the donor information access process does not apply (unless their donor has chosen to remove their anonymity)^65^; and (iii) those born of anonymous donations made prior to 1 August 1991, whose information is not stored by the HFEA and to whom the donor information access process does not apply.

Two key issues arise as a result of the operation of the donor information access process to exclude pre-2005 donor-conceived people, which we consider below from the perspective of procedural justice. The first is the denial of their Article 8 right to information about their donor, the second, the consequence of the first for many donor-conceived people, is the impact of the use of DTCGT to search for that information. Thinking back to Dworkin’s characterization of ‘moral harm’, we suggest that the denial of the Article 8 right to pre-2005 donors (and to post-2005 donors during the first 18 years of their life) opens up the possibility of their suffering the ‘moral harm’ of shock discoveries about their identity, whether as a result of their use of DTCGT or otherwise. There is a risk of similar harm being suffered by others who are ‘discovered’ by donor-conceived people from all cohorts searching for their donor, or relatives through donor conception (donor relatives). We turn first to the role of the donor information access process in denying donor-conceived people access to information about their origins.

### C. Donor anonymity and the importance of information about genetic origins

The decisions in *Rose* and *Odièvre* established the significance of information about their genetic origins for someone’s sense of identity. This has also been recognized in the context of adoption, where an adopted person is entitled to access their birth mother’s identity should they choose to do so.^66^ However, for pre-2005 donor-conceived people this right is displaced by the guarantee of anonymity made to their donor. Many donor-conceived people fundamentally disagree with this approach:

My personal view is that, that nobody has the right to erm, to create a treatment to bring a child into the world where they can’t know whose eyes they’ve got, or whose hair they have, what illnesses they might be carrying…there really aren’t many words you can use to describe how wrong it is. [Patricia, donor-conceived person (DCP)]

I have [the] same right as everyone else—that is to know who my biological father is. And, you know, the fact that my parents made some weird arrangement…I don’t feel any sympathy for my biological father really. I mean, I didn’t agree to him being anonymous…My family history’s been hidden from me my whole life… I’ve done nothing wrong. I’m just to trying to find out who I am [Fiona, DCP]

That the donor information access process continues to protect the anonymity of donors evoked strong responses in some of our participants:

I think that it’s sort of—a large part of it is because even now, they don’t think of them as human beings…they’re like products that are produced…So, I think there’s an element of that in the HFEA. [Cleo, parent of DCP]

I do think the HFEA and eventually the Government are just going to have to go back to you know back to the beginning and re-think, they really are. [Emily, parent of DCP]

This was the case even for identity-release donors whose information would, pursuant to the donor information access process, be available to those born from their donation from the age of 18 years:

I think anonymity is wrong, plain and simple…And I think identity release is a form of anonymous because of what it does to someone who’s DC—they have no information until they reach 18, so the formative years are gone…if one DC person is suffering and there’s no…laws in place to help them on their journey of identity…then I think that’s one too many. [Anita, DCP]

Turning back to the theories of procedural justice summarized above, we can see that, from a relational perspective, the donor information access process falls short in various ways. First, by creating cohorts with different rights to information, the process fails to treat donor-conceived people fairly or even-handedly. In the context of information which enables someone to establish the details of their identity as a human being this is significant, particularly because respect for someone’s private and family life pursuant to Article 8 incorporates the concept of personal identity.^67^ Secondly, according to Galligan’s description of procedural rights, the donor information access process, in failing to protect the Article 8 rights of donor-conceived people, does not take them seriously, denying donor-conceived people, and especially pre-2005 donor-conceived people, the dignity, agency and autonomy which respect for their Article 8 rights would afford them. In restricting access to information, which is likely to be understood as constitutive of someone’s identity, the donor information access process removes the choice that their Article 8 right offers to understand this aspect of themselves. While post-2005 conceived donor-conceived people are unable to access this information until they are 18 years, in mandating the offer of implications counselling,^68^ the donor information access process seeks to ensure that, when it does become available to them, information is provided in a ‘safe space’ which acknowledges its significance.^69^

Without access to the ‘safe space’ created by the donor information access process, the only realistic choice available to many donor-conceived people is to use DTCGT to search for information about their donor. However, in combination with the culture of secrecy surrounding donor conception (particularly with respect to pre-2005 donor-conceived people whose donors will always have donated anonymously) and the very real possibility that many donor-conceived people will be unaware of the circumstances of their conception, the use of DTCGT comes with significant risks.

### D. DTCGT and the slow violence of keeping genetic secrets—‘a time bomb ticking away’^70^

It has long been acknowledged that allowing children to grow up believing untruths about the circumstances of their birth has the potential to harm them if the truth unexpectedly comes to light. In the context of adoption, for example, the Tomlinson Report, published in 1925, questioned whether it is, ‘desirable or even admissible deliberately to eliminate or obscure the traces of a child’s origin so that it shall be difficult or impossible thereafter for such origin to be ascertained’,^71^ and, in 1953, the Hurst report, considering law reform in the context of adoption, went even further:

We have been told of tragic cases where the child has learned the truth [about having been adopted] suddenly from strangers with disastrous psychological consequences. In some cases children, especially in adolescence, have become mentally unbalanced from the shock. We were much impressed by the evidence we have received about the great importance of the child being brought up from his earliest years to know he is adopted.^72^

Thus, the importance to someone’s sense of self and identity of the story of their conception and (in the case of adoption) their early life, has been recognized for nearly a century. As we have seen, however, the Warnock Committee gave priority to the perceived benefits of anonymity for donors and recipients (and the interests of the nascent fertility industry in not putting potential donors off).^73^ For those donor-conceived people who remain unaware of the circumstances of their conception,^74^ the trauma anticipated by the Hurst report might, as a result of DTCGT, become their reality. This was the experience of some ConnecteDNA participants:

I didn’t want to believe it and[…]then I thought what if my mum had an affair, so there was a lot of confusion and space and time kind of warped around that time, yes. And yes, it was very odd, very painful, and I started suffering from what I thought was shock, but now that I’m in therapy, I realise it was what they call PTSD and also CPTSD, I think that’s complex PTSD[…]it’s a different form of PTSD, it’s linked to, like, specific events in life, and they all accumulate and come and find you later on. I was displaying symptoms of[…]PTSD, so I couldn’t complete my work[…]and because you know, the layers were starting to hit, the waves of what happened, what really happened, I couldn’t perform to the best of my abilities and the people that hired me didn’t understand what was happening, so they thought I wasn’t what I showed in the interview and the training so they were like, “Sorry, we can’t keep you on.” [Anita, DCP]

With this in mind, we now turn to Nixon’s concept of ‘slow violence’ to explore the damaging potential of DTCGT for those affected by donor conception.

Nixon writes thought-provokingly about ‘slow violence’ in the context of environmental catastrophes, such as climate change, which, although predictable (and predicted) are not immediately ‘dramatic enough to rouse public sentiment and warrant political intervention’.^75^ He characterizes slow violence as, ‘a violence that occurs gradually and out of sight, a violence of delayed destruction that is dispersed across time and space, an attritional violence that is typically not construed as violence at all’.^76^ He contrasts violent events which are ‘immediate in time, explosive and spectacular in space’ with those which are ‘incremental and accretive, [whose] calamitous repercussions [play] out across a range of temporal scales’.^77^

Barnwell applies Nixon’s notion of slow violence to the temporal dimensions of social problems, because it addresses ‘unseen structural forces that sustain social injuries and protect them from exposure’.^78^ She focuses on families’ management of potentially stigmatizing information by secrecy, often across generations, noting that secrets are conditioned by historical, social, and political processes, which direct codes of conduct and silence, but often change over time. She suggests that families, ‘experience a double form of violence, where the risk of social stigma pressures the family unit into silence and then, in a second blow, leaves subsequent generations to deal with the often hurtful or confusing legacy of secret-keeping’.^79^

Our data evidence this in the context of donor conception, revealing a spectrum of hurt that ranges from extreme:

I just couldn’t, you know, I didn’t speak to [my mother] her for a couple of weeks. And I couldn’t look at my face in the mirror. And I just, I couldn’t think about anything, do anything other than think about what the hell has happened. It felt like my whole childhood was a lie. And every time I thought about my grandparents on my dad’s side who I was very close to, I just couldn’t, I’d break down in tears. Because obviously they didn’t know and[…]would they have loved me if they’d have known? [Fiona, DCP]

to a feeling of sympathy with the predicament of a parent who, due to societal pressures, did not feel able to disclose the fact of donor conception:

So, anyway, I just, I got my parents in a room and I said, “What has happened?” And they said[…] “When we went for tests and things we discovered that, you know,[…] dad’s sperm wasn’t viable. So, we used a sperm donor. The hospital used a sperm donor.”[…] And I was obviously really shocked but also like, I don’t know, it just became more about my dad at that point. Like he just, I’ve never seen him cry before and he just broke into tears and it, it was just horrible. It was like, I had to look after him. [laughter] Even though it was a secret about me. And, you know, they were obviously just heartbroken and, and, I dunno. It was weird for me but I wasn’t, I wasn’t mad at them for keeping a secret, you know. They’d done a really good job of it because people have always told me I look like my dad. [laughter][…]And they were just like, “We had no idea that these websites were gonna, you know, DNA testing would be a thing in the future. Nobody knew apart from us two and the hospital. Like literally no one else knew that we did this.” [Lisa, DCP]

Further, for some parents of donor-conceived children, there is an awareness of potentially i*nstigating* a type of slow violence by choosing to use DTCGT to search for their child’s donor relatives when they are too young to take any part in decisions but will inherit the consequences of them. While that is to some extent true of all the decisions we make for our children, the legal protection of anonymity in the context of donor conception distinguishes these circumstances from more ‘run of the mill’ decisions:

And it’s just that, still this balance of, does [child] turn round to me in 10 years, and [say], “Oh, you,” you know, “You gave away my DNA,” versus, “Oh, you could have done a DNA test and I could have found my genetic family earlier.” So, I mean I feel the weight of that (Faye, parent of DCP).

Across our dataset, and thinking specifically about anonymity and information sharing, there is a clear recognition that the interests of donors, donor-conceived people and parents vary, and that tensions exist which are difficult (and may be impossible) to resolve. However, for many, the donor information access process is out of balance in its continued prioritization of the interests of donors (and, arguably, those of the fertility industry) over, and to the detriment of, those of donor-conceived people.

Looking back to the Warnock Report, it is clear that the Committee’s intent was to balance the various interests it had identified, and that, at that time, protecting donors’ identity was considered the best way to proceed.^80^ A different balancing act was undertaken in *Odièvre v France*, where the applicant was denied access to identifying information about their birth mother. It is particularly interesting for our purposes here to look at the joint dissenting opinion,^81^ where the Judges felt that an appropriate balance between the competing rights and interests *had not* been achieved:

That is the nub of the problem…no balancing of interests was possible in the instant case. … French law accepted that the mother’s decision constituted an absolute defence to any requests for information by the applicant…the mother’s refusal is definitively binding on the child, who has no legal means at its disposal to challenge [it]. The mother thus has a discretionary right to bring a suffering child into the world and to condemn it to lifelong ignorance[…]The effect of the mother’s absolute “right of veto” is that the rights of the child…are entirely neglected and forgotten. In addition, the mother may also by the same means paralyse the rights of third parties, in particular those of the natural father or the brothers and sisters[…]

Even if it has been adopted, a child who is unable to gain access to any type of information about its family origins is made to endure a form of suffering, and that suffering may leave scars.^82^

Returning to the donor information access process, and reflecting on the moral significance, in the relational account, of social marginalization and inequalities of social power and standing, we can understand how those affected by donor conception might feel about being left with no choice but to turn to DTCGT in the search for information about their donor. Participants in our stakeholder workshop, for example, while acknowledging the risks of DTCGT use, felt that it was the statutory donor information access process and the HFEA which had let them down:

[DCP] shouldn’t have to do a DNA test to find biological relatives. Online DNA testing is not the problem just there is no other way. The regulated sector should limit the power of DNA testing to impact DC people. [Stakeholder workshop ‘world café’ discussion]^83^

Further, and thinking specifically about the potential for DTCGT being the catalyst for harm in terms of the exposure of family secrets, we can also see the relevance of the relational account of agency around which Meyerson and MacKenzie’s model of procedural justice is constructed. Participants, whose use of DTCGT to find their donor leads them to identify other relatives by donor conception, for instance ‘donor siblings’ born from the same donor’s donation(s), must decide whether to explain the nature of the genetic connection or not, considering that their donor sibling may be unaware of the circumstances of their conception. We see in practice the social, embodied and emotional exercise of agency that Meyerson and MacKenzie describe, as donor-conceived people become ‘gatekeepers’ of others’ secrets and have to make ethically challenging decisions in their search for information. Turning to Rundle’s characterization of legal processes as demonstrating an authority’s commitment to lawful authority rather than ‘mere power’, we suggest that, in constraining the choices, removing ‘safe’ decision-making spaces and acting as a barrier to the rights of many donor-conceived people, the donor information access process acts on them in a powerful sense, rather than engaging with them through the medium of a just procedure, thus conveying indifference both to their right to self-determination and to their well-being.

For these reasons, we argue that the donor information access process is flawed, and that in denying donor-conceived people dignity, rights, and agency, it is intrinsically unjust. For these reasons, reform of the 1990 Act is needed. By reference to the HFEA’s consultation process on reform, we now consider what is required to more fairly attend to the rights and interests of all those affected by donor conception.

## IV. MOVING ON FROM DONOR ANONYMITY: AN EVIDENCE-BASED RESPONSE TO THE HFEA’S PROPOSALS FOR REFORM

It is clear that DTCGT brings a new dimension to the regulation of donor conception. For some, this is positive but for others, especially those for whose family life has been impacted by an unexpected result, (whether theirs or someone else’s) it is not. DTCGT thus has the power to energize the slow violence of the secrets the system has encouraged people to keep, with destructive potential. In addition, the use of DTCGT by parents of donor-conceived children potentially instigates a new current of slow violence, in that, when they reach an age at which they are able to decide for themselves, those donor-conceived people might take a different view from their parent(s) about sharing their DNA. In the past, in shaping the legal and regulatory framework, regulators, and clinicians (although aware of the experiences of adoptees) were constrained by a lack of understanding of the constitutive importance for donor-conceived people of information about their donor and donor relatives. A growing body of research means this is no longer the case. The disruptive effect of DTCGT on the donor information access process offers an opportunity to shape a law reform process that, in a procedurally just manner, seeks to review and re-prioritize the rights of donor-conceived people to access information about their donor, and those of historic donors to continuing anonymity.

Access to donation information was one of the four issues around which the HFEA’s 2023 consultation about modernizing the regulation of fertility treatment was arranged. The consultation document made specific reference to the disruptive effect of DTCGT:

The issue of accessing donor information and identifying donors has become more urgent with the growing popularity of easily accessible, relatively affordable direct-to-consumer DNA testing and matching services[…] These services can provide information to those who previously had no way of finding out their full genetic origins.^84^

The timing of the consultation, and the convening of the HFEA’s Legislative Reform Advisory Group (LRAG), enabled us to discuss the proposals for reform of the 1990 Act with stakeholder workshop participants. The LRAG’s comprehensive discussion document, ‘Donor anonymity and information provision’ offered four ‘initial thoughts on possible options for a new model’; status quo plus, early identification by consent, removal of anonymity completely, with increased emotional support for all parties, and a ‘double track’ system, in which donors would choose between the status quo (ie, becoming identifiable at 18 years) or being identifiable from the outset.^85^

Underpinning the status quo plus model was the assumption that some of the reasons for maintaining donor anonymity until the donor-conceived person reached the age of 18 years remained relevant. The ‘plus’ element referred to a proposed legal obligation for clinics to inform donors and recipients about the risk that, as a result of DTCGT, their identity might be discovered earlier than that. Our participants were generally unconvinced by this proposal. They felt that the retention of anonymity was both unattractive and, in light of the existence of DTCGT, impractical, although the importance of informing donors and recipients of the potentially disruptive effect of DTCGT on donor anonymity was acknowledged.

The ‘early identification by consent’ model anticipated the introduction of a voluntary system for donors to become identifiable before any children born of their donation reached the age of 18 years, perhaps in conjunction with ‘localised arrangements’ about, for instance, the level of contact required and at what point the child might be involved in decisions. It was noted that information might still emerge from other sources. While early identification (in the context of donor agreement) was supported by workshop participants, the fact that anonymity would remain the standard for those who chose not to opt for early identification was unpopular for the reasons stated above. Further, participants felt it inappropriate that the decisions would be left in the hands of donors and parents because, ‘consents between donors and parents [do] not account for the welfare of the child’ [World café discussion, Manchester workshop].

Removal of anonymity completely was the most popular option, with this being described as ‘the ideal minimum standard’ and ‘the most considerate action for the child’ [World café discussion, Birmingham and Manchester workshops, respectively] The LRAG considered how law and regulation might respond, including whether a reformed legislative framework should apply retrospectively, as has happened in Victoria, Australia.^86^ Workshop participants expressed a variety of opinions as to the retrospective removal of anonymity. Some were strongly of the view that retrospective change should happen, with one parent at the London workshop specifically noting that the ‘Victoria model would be very attractive’ to her daughter (conceived from a pre-2005 donation). However, others were in favour of prospective change to protect historic anonymous donors, with some of those suggesting that removal of anonymity should be accompanied by a change to the birth certification arrangements to ensure that information would be available to donor-conceived people whether or not their parents had told them about the circumstances of their conception.

The final possibility was a ‘double track system’, in which donors would choose between the status quo (being identifiable after the child reached the age of 18 years) and being identifiable from the outset. A double track system, it was suggested, might offer more autonomy to donors and patients, in deciding what information (or contact) they wanted, and when although, it was noted, would fall short of offering the same level of autonomy for donor-conceived people. And, of course, that DTCGT would make donors’ ‘choice’ to opt for anonymity unrealistic. Our participants were again divided. Those in favour felt that choice was a benefit, but those against felt that such a system would still disadvantage the child (who would not have a choice) by continuing to vest power over this information in the hands of others.

### A. The ConnecteDNA response to the HFEA’s 2023 consultation

The variety of options tabled in the LRAG discussion document was not reflected in the HFEA’s consultation, which essentially only sought views on the ‘double track’ option. The question respondents were asked, in relation to donor anonymity, was whether:

the [1990] Act should be amended to provide parental and donor choice to opt for anonymity until age 18 (as now) or identifiable information on request after the birth of a child?^87^

Our stakeholder workshop discussions enabled us to offer a response to the HFEA’s consultation which directly reflected the perspectives of participants in our research. As noted above, some attendees liked the choice this option would provide, while others felt that, despite allowing flexibility, having a variety of options might cause confusion. There was a general feeling that, with the availability of DTCGT, a donor’s identity can no longer be protected, so the ‘dual track’ option would not be practical.^88^ Further, participants raised concerns about the potential for inequalities either to be created or exacerbated by a ‘dual track’ system, noting that donor-conceived people would have different ‘rights’ under any dual track system, depending on the basis on which their donor consented. Some were concerned that fertility clinics, being commercial entities, might start charging more for donors who had consented to be identifiable from birth.

While, in general, participants felt that the ‘dual track’ approach proposed would be preferable to the existing legal position, our data suggested that an agree/disagree proposition was insufficiently nuanced. Rather, our research indicates that donor-conceived people’s views, experiences and circumstances are varied and change over time. Parents (even when they have had good implications counselling) can often only fully engage with the implications of their child having (or not having) information about their donor or donor siblings, once the child exists, starts to express views and to develop needs and interests. Several parents who took part in the ConnecteDNA study told us that their interest in using DTCGT was, at least in part, motivated by feelings of guilt about not having used a known donor to conceive. Others sought to identify donor relatives in response to health concerns, even minor questions about a child’s allergies, as well as to respond to their child’s growing curiosity. Thus, our response to the consultation proposed a more flexible system. Such a system would enable parents with a donor-conceived child (of any age) to request information about their donor if and when needed, and would allow the donor to consent to (or refuse) contact based on their circumstances at the time. It would also facilitate some form of mediated communication for those who did not desire identifying information, as well as enabling the exchange of identifying information (by mutual consent) for others.

In terms of access to information, the HFEA’s proposal made reference only to information about the donor. Our research found that connections with same-donor siblings are often seen as equally (and, sometimes more) important, to donor-conceived people than contact with their donor. Indeed, the desire to connect with same-donor families and siblings during a donor-conceived person’s childhood is a key driver for the use of DTCGT, it being the only option available to facilitate those connections. However, the use of DTCGT in this way, as discussed above, carries the risk of crystallizing the ‘slow violence’ of previous secrecy where connections are made with people who do not know that they are donor conceived, with gamete donors who want to remain anonymous, or with anonymous gamete donors’ family members.

Conscious of the difficulty in attending to the interests of all those for whom reform of the 1990 Act might be relevant, we suggest that a principled approach offers a mechanism to ensure a procedurally just process.

## V. MOVING ON FROM DONOR ANONYMITY—A PROCEDURALLY JUST PROCESS OF LAW REFORM

Our interest in procedural justice is less concerned with people’s willingness to comply with laws and legal processes and more related to the relational reciprocity that Rundle suggests underpins the distinction between acting on people with ‘mere power’ and exercising lawful authority. Compliance with legal rules is tangentially relevant, particularly to the post-2005 cohort. A growing number of parents of post-2005 donor-conceived people are using DTCGT to search for their children’s relatives.^89^ They acknowledge that, in using DTCGT for their children under the age of 18 (the age below which most DTCGT sites terms and conditions theoretically prohibit access to their genetic testing services^90^) they are disregarding a rules-based framework and, in so doing, are also choosing to circumvent the donor information access process. This is despite the fact that using DTCGT can entail difficult moral choices. The 1990 Act, in preventing the access to information that DTCGT can offer, thus has power but conveys the indifference to donor-conceived subjects’ agency and the rights to identity-building and self-determination that Rundle describes (and Article 8 protects). For the parents of donor-conceived children, the impact is on their agency to facilitate the sibling and other familial relationships they consider will benefit their children as they grow up. For this reason, on Rundle’s analysis, the 1990 Act lacks both legal and moral authority.

To achieve procedural justice, we suggest a principled approach to reform of the 1990 Act. The principles around which we base our argument reflect the priorities discussed by our participants but also acknowledge that the rights and interests of donors, particularly historic donors who were promised anonymity, are often not aligned with those of donor-conceived people. In selecting these principles, we are using a precedent for reform set by the Australian state of Victoria.^91^

### A. A principled approach to reforming the 1990 Act

It is clear that the use of DTCGT ‘matching’ and ‘relative finder’ databases, in conjunction with social media and other publicly available databases of information, allow donor-conceived people in many cases to trace and, often, to contact their donor and/or donor-relatives. The consequence of this occurring outside the donor information access process is that regulatory protections, such as being offered access to counselling (or information and support services), are not available. Accordingly, our contention is that the goal of reforming the 1990 Act should be to provide a framework to ensure that information exchange, and any contact, occurs in a protective and appropriate manner, with a supportive system for all affected by donor conception. We suggest that, to achieve this, consideration must be given to the removal of donor anonymity with retrospective effect, as has been the approach in Victoria, Australia.^92^

Assuming that a broadly drawn participatory inquiry were to take place, its findings could inform an evidence-based set of principles to inform legislative reform. Our data, and the responses to the HFEA’s consultation, suggest that the five principles which underpinned the approach of the Victorian state government to legislative reform in this area might also be helpful here.^93^ These principles assume that:

1. The law should treat all donor-conceived people consistently and equally, regardless of when the donations that led to their conception were made.
2. Knowledge of their genetic identity is critical to the welfare and interests of donor-conceived people.^94^
3. The impact of releasing identifying information on donors, their wider families, and donor recipient parents should be considered.
4. The rights conferred by the law on donor-conceived people should be meaningful and, as far as practicable, exercisable.
5. Legislation should not place undue regulatory burdens on medical practitioners and health services.^95^

We have described above (and, indeed, the 2004 Regulations acknowledge) the importance of the second principle. The discussion that follows concentrates particularly on Principles 1 and 3.

### B. Principle 1: consistent and equal treatment of all donor-conceived people

That the law should treat people consistently and equally, as demanded by Principle 1, seems uncontroversial. However, we have described how the 1990 Act, by the donor information access provisions, creates cohorts of donor-conceived people with very different rights to the information which, by reference to Principle 2, is critical to their welfare and interests. Our contention is that this is procedurally unjust, and that the HFEA’s recent proposals for reforming the 1990 Act will exacerbate the inequity further.

The HFEA published its proposals in November 2023. It concluded that, in effect, the availability of DTCGT ‘matching’ databases and social media had created an informal ‘dual track’ system in the UK and, thus, that the status quo was no longer sustainable. For these reasons, the HFEA recommended the (prospective) removal of donor anonymity from the birth of any donor-conceived child and, hence, no double track system.^96^ Anticipating in-depth discussions with patient and donor groups, donors and donor-conceived individuals, and licensed centres within the fertility sector, the HFEA noted that its proposal on the removal of anonymity was subject to further consideration of certain points. These included the position of both pre- and post-2005 donors. The wording in the HFEA’s document seems, however, to suggest that retrospective removal of anonymity is not anticipated:

The HFEA’s proposal on the removal of anonymity is subject to consideration on the following points:[…] Continued respect of donor anonymity for pre-2005 donors and no retrospective early removal of anonymity for post-2005 donors.^97^

If the HFEA’s recommendation is reflected in further prospective reform of the 1990 Act, another cohort of donor-conceived people will be created, again with different rights. The inequities and procedural injustices we have described above will remain a feature of the donor information access process: pre-2005 donor-conceived people will continue to have to turn to DTCGT to search for information outside of any ‘safe’ statutory process and those born of donations made after 2005 but before the date of the prospective change will still have to wait until they are 18 years to access information from the HFEA. This is despite the fact that the importance of information about genetic identity has been recognized by law- and policy-makers since the Tomlinson Committee’s deliberations in 1925. Indeed, by the time the Warnock Committee was convened, Parliament had passed into law, a piece of legislation that, with retrospective effect, provided for all adopted people, on reaching the age of 18 years, to have access to information about their birth mother.^98^ The implications for the birth mothers who had been guaranteed anonymity were considered, but the constitutive importance of their birth records for ‘the many people who wish to complete their process of self-identification’ held sway.^99^

However, the HFEA’s recent proposals are a crucial line in the sand. Irrespective of the fact that this call for change is likely to have been catalyzed by the disruptive effect of DTCGT on promises of donor anonymity rather than a concern for the plight of those without access to the ‘constitutive’ information they seek, change is coming. Our call here is for a process of change that is attentive to the demands of procedural justice in the context of an issue which cannot be divested from the relational implications of information and identity. It is clear that the HFEA intends to inform any proposed change to the law with in-depth and inclusive discussions^100^ and, to that extent, attention will be paid to the fundamental importance of the participatory practices which sit at the core of a relational account of procedural justice. A key facet of the process after the participatory phase(s), however, will be a consideration of the rights and interests of *all* donor-conceived people, which have diverged widely over time. To achieve consistency and equality, to restore the internal morality of the regulation of donor conception, and to give procedural effect to donor-conceived people’s Article 8 rights, the legal implications of these temporal dynamics require harmonization. In our view, this requires an evaluation of the harms and benefits of removing donor anonymity with *retrospective effect*. The rights of historic donors to anonymity have, to date, been accorded great weight in decision-making about access to donor information. However, the disruptive effect of DTCGT means, as the HFEA has recognized, that for future donors, promises of anonymity are no longer wholly tenable. The same is true for historic donors (and their families). Simply put, the status quo is (potentially) detrimental to all.

### C. Principle 3: the impact, on donors and others, of releasing identifying information

In our discussion above, we have argued that, from the perspective of donor-conceived people, the current donor information access process is unjust. In considering the retrospective removal of donor anonymity, it is to the rights and interests of donors that we now turn.

Donors’ expectations of anonymity are grounded in the circumstances and agreements that applied when they donated their gametes. It is likely, in all cases, that donors were assured that their anonymity would be protected and that they relied on those assurances. Donors who participated in the ConnecteDNA research expressed a range of opinions when asked about the removal of anonymity, with some being strongly opposed:

I wouldn’t agree with forcibly removing my anonymous data. I wouldn’t agree with that, no.[…] I think forcibly doing that without your consent would be wrong, because you entered into an agreement. Consent’s very important. … Makes me feel violated. [Howard, donor]

and others taking a very different view:

I do think everyone has a right to know, biologically and DNA wise where they’ve kind of started really…That doesn’t mean that there has to be any expectations of what that relationship might look like in the future. [Nia, donor]

In considering how to approach a weighing of the rights and interests of donors in retaining their privacy and donor-conceived people accessing information about their biological parent, there were similar divergences of opinion:

We seem to be in a situation now where the rights of unborn child somehow seem to be taking on a new kind of salience it didn’t have, say, 20, 30 years ago. … but I really don’t see how someone who isn’t even born can have rights, legal rights. [Will, donor]

I have [the] same right as everyone else that is to know who my -biological father is. And, you know, the fact that my parents made some weird arrangement…I don’t feel any sympathy for my biological father really. I mean, I didn’t agree to him being anonymous…My family history’s been hidden from me my whole life…I’ve done, I’ve done nothing wrong. I’m just to trying to find out who I am. [Fiona, DCP]

I feel like children have some kind of rights to understand their background but at the same time if somebody has donated under the terms of it being anonymous then you can’t really go back on that. It’s a tricky one … The tricky thing is of course that the child is not part of that contract. [Simon, donor]

A range of legal questions would have to be addressed in considering whether the retrospective removal of donor anonymity would be appropriate, including the implications for medical confidentiality and privacy law more generally, the importance to be accorded to principles of contract law and the significance of donations having been made in reliance on the assurance of anonymity, particularly as regards the proportionality of relying on a contractual term or condition to defeat a substantive Article 8 right. However, whatever the outcome of any participatory process set in train to examine these issues, it seems undeniable that an evidence-gathering process, based on evidence from across the spectrum of relevant opinions and experiences, is essential. A failure to consider the merits and disadvantages of a retrospective removal of anonymity based on such a process would appear straightforwardly to prioritize the contractual rights of historic donors over the present distress of many people affected by donor conception. These would include not only the donor-conceived people who would continue to be excluded from the donor information access process, but also those donors and their relatives (by donation and otherwise) identified as a result of DTCGT on whom the slow violence of historic practices of secrecy may then act. This, surely, would render the process procedurally unfair.

### D. Principles 4 and 5: Achieving meaningful rights and avoiding undue regulatory burdens

To the fourth principle, it is clear that, in practical terms, the implications of a retrospective change would be straightforward for 1991–2005 donors, whose information is held by the HFEA, but not for those who donated earlier. Before 1991, no centralized records were kept and it will be challenging both to engage historic donors and to confer meaningful and exercisable rights on those conceived before the 1990 Act came into effect. However, that is not a reason to maintain the status quo in respect of pre-1991 donations. DTCGT might have to become a tool in the HFEA’s future toolkit in the event that no official information is available and we suggest that counselling and support should be available. In our view, the scope of the law reform project should encompass legislation to guarantee (funded) support and counselling, on request, for all those affected by donor conception on whose lives and relationships DTCGT has impacted and, possibly, a licensing scheme so that DTCGT providers contribute to that cost. We would argue that web-based materials and information, with which the HFEA proposes to replace the funded support and intermediary services currently available, are insufficient.^101^ In our view, it is arguable that, for those applying for information pursuant to the donor information access provisions, publishing web-based information and signposting to counselling services falls short of the statutory obligation to offer a suitable opportunity to receive access to proper counselling.^102^

Clearly a law reform project of this nature will be labour- and resource-intensive. However, we note once more Meyerson and MacKenzie’s assertion that attendance to relational dynamics as a core element of procedural justice is essential, even where resources are limited. Attention to the rights and interests of all of those on whom a particular legal instrument will act is a fundamental requirement that cannot simply be balanced away in the service of timeliness and efficiency.^103^

## VI. CONCLUSION

In discussing and evaluating the donor information access process, we have suggested that no continued normative basis for the protection of donor anonymity exists. Indeed, *Rose* has established that access to information about genetic origins is within the scope of Article 8. However, practices of secrecy in relation to donor conception, including the promise of anonymity to historic (largely sperm) donors, and the way in which the 1990 Act has been reformed since it came into force, have combined to create legal impediments to the removal of donor anonymity. At the same time, the availability of (relatively) affordable DTCGT, and the complex combination of benefits and ‘slowly violent’ harms which can result from its use by those affected by donor conception, have driven a coach and horses through the existing statutory framework. The HFEA has acknowledged that the disruptive effect of DTCGT has undermined the integrity of the 1990 Act, and that reform is required.

We have suggested that, to ensure a procedurally just process of reform, consideration of the rights and interests of all those affected by donor conception is required, including those who donated before the 1990 Act came into effect. We have also suggested that, as a matter of principle, procedural justice would require that all donor-conceived people should be treated equally under the 1990 Act. Equal treatment for all donor-conceived people requires that the same donor information access process should apply, irrespective of the date on which the donation which led to an individual’s birth was made.

This means that, whatever approach to donor anonymity is considered most fairly to represent the rights and interests of all those affected by donor conception (including all cohorts of donors and donor-conceived people and their families), *it should be the same for all donor-conceived people*. Thus, whatever the outcome of the participatory evidence-gathering and wider legislative process, even if the *status quo* is to be maintained, reform of the donor information access provisions with retrospective effect will be required.

## Footnotes

1 Department of Health & Social Security, *Report of the Committee of Inquiry into Human Fertilisation and Embryology* (Warnock Report) (Cmnd 9314, 1984) para 20.

2 The direct-to-consumer genetic testing and donor conception: support and governance in emerging digital systems (ConnecteDNA) research is funded by UKRI’s Economic and Social Research Council (ES/V002430/1) and led from The University of Manchester by Professor Lucy Frith. Our paper, Direct-to-Consumer Genetic Testing and the Changing Landscape of Gamete Donor Conception: Key Issues for Practitioners and Stakeholders’ (2023) RBMO <doi.org/10.1016/j.rbmo.2023.103421> provides in full the methodology of the study: recruitment, sample size, limitations, and analytic methodology.

3 Human Fertilisation and Embryology Act 1990, s 31ZA.

4 By this we mean genetic connections through their gamete donor. ‘Donor siblings’ would include, for example, other people conceived from the donations of the same donor as well as that donor’s ‘social’ family.

5 Human Fertilisation and Embryology Act 1990, s 31ZA(3)(b). Note, however, that in January 2024 the HFEA decided to withdraw funding for counselling services, which are likely to be replaced with web-based information and support. See <www.hfea.gov.uk/media/gr4pkbtr/2024-01-24-minutes-of-authority-meeting.pdf> accessed 29 May 2024.

6 Please see our article, ‘Direct-to-Consumer Genetic Testing and the Changing Landscape of Gamete Donor Conception: Key Issues for Practitioners and Stakeholders’ (2023) RBMO <doi.org/10.1016/j.rbmo.2023.103421> for further information about the qualitative study.

7 Anthony Bottoms and Justice Tankebe, ‘Procedural Justice, Legitimacy and Social Contexts’ in Denise Meyerson, Catriona Mackenzie and Therese MacDermott (eds), *Procedural Justice and Relational Theory* (Routledge 2021) 85.

8 Home Office *Child Adoption Committee First Report* (Cmnd 2401, 1925) (Tomlinson); Home Office *Report of the Departmental Committee on the Adoption of Children* (Cmnd 9248, 1953) (Hurst).

9 We illustrate our argument with quotes from ConnecteDNA project participants. Participants real names are not used, and quotes have been ‘cleaned up’ to aid readability.

10 Human Fertilisation and Embryology Act 1990, s 31ZA. Note that donor-conceived people can access non-identifying information about their donor at the age of 16 years.

11 Rob Nixon, *Slow Violence and the Environmentalism of the Poor* (Harvard University Press 2011).

12 Ashley Barnwell, ‘Family Secrets and the Slow Violence of Social Stigma’ (2019) 53 Sociology, 1111.

13 Nixon (n 11) 3.

14 ibid.

15 Barnwell (n 12).

16 HFEA, ‘Modernising the Regulation of Fertility Treatment and Research Involving Human Embryos’ www.hfea.gov.uk/media/y3xdctuq/modernising-the-act-consultation.pdf >accessed 30 January 2024.

17 Law Reform Committee, Victorian Government, *Inquiry into Access by Donor-Conceived People to Information about Donors* (Parliamentary Paper No 120, Victorian Government, Session 2010-2012).

18 Warnock Report (n 1) para 4.

19 ibid para v.

20 ibid.

21 ibid.

22 1990 Act, s 31(2).

23 The Human Fertilisation and Embryology Authority (Disclosure of Donor Information) Regulations 2004, SI 2004/1511 (2004 Regulations).

24
*Rose v Secretary of State for Health and Human Fertilisation and Embryology Authority* [2002] EWHC 1593 (Admin).

25 Council of Europe Treaty Series 005, Council of Europe, 1950.

26 Department of Health. *Donor information: Providing information about gamete or embryo donors:*
*Responses to consultation*. 28 January 2003. For a discussion see Lucy Frith, ‘The limits of evidence: evidence-based policy and the removal of gamete donor anonymity in the UK’ (2015) 33 Monash Bioethics Review 29.

27
*Odièvre v France* [2003] 38 EHRR 871.

28 ibid [44].

29 2004 Regulations, Regulation 2(3). Ss 30-31ZG were inserted into the 1990 Act by the Human Fertilisation and Embryology Act 2008. Donor-conceived people may, from the age of 16, apply for non-identifying information about their donor, such as height, weight, ethnicity, marital status, and relevant personal/family medical history. 1990 Act s 31ZA(2)(a) and (4).

30 See the HFEA’s website for further information: <www.hfea.gov.uk/donation/> accessed 2 November 2023.

31 HFEA (n 16) 15.

32 See <www.ancestry.co.uk/> and <www.23andme.com/en-gb/> respectively, accessed 17 October, 2023.

33 Including, for example, ancestry services, health services such as health predisposition reports, carrier status reports, heart health reports, migraine reports. See <www.23andme.com/en-gb/> accessed 2 November 2023.

34 See eg, ‘It Started with Her Name’: The Backstory to Ancestry’s New TV Ad Series’ <https://blogs.ancestry.co.uk/ancestry/2021/03/03/it-started-with-her-name-the-backstory-to-ancestrys-new-tv-ad-series> accessed 17 October 2023.

35 See our article, ‘Direct-to-Consumer Genetic Testing and the Changing Landscape of Gamete Donor Conception: Key Issues for Practitioners and Stakeholders’ (2023) RBMO <doi.org/10.1016/j.rbmo.2023.103421> for further discussion.

36 In relation to which, the methods, findings and conclusions are in our article, ‘Direct-to-Consumer Genetic Testing and the Changing Landscape of Gamete Donor Conception: Key Issues for Practitioners and Stakeholders’ (2023) RBMO <doi.org/10.1016/j.rbmo.2023.103421>

37 These workshops took place in London, Manchester and Birmingham in the autumn and winter 2022/2023. An open invitation was issued to stakeholder communities. Between 10 and 20 people participated in each.

38 Including reproductive medicine clinicians; members of the British Infertility Counsellors Association; academics with expertise in the area; members of the Donor Conception Network; representatives of Donor Conceived UK.

39 HFEA, ‘Donor anonymity and information provision’ (27 May 2022) <www.hfea.gov.uk/media/nacb35fx/lrag-discussion-paper-donor-anonymity-and-information-provision-2022-05-27.pdf> accessed 30 January 2024.

40 Warnock Report (n 1).

41 Denise Meyerson and Catriona MacKenzie, ‘Procedural Justice and the Law’ (2018) 13 Philosophy Compass. <https://compass.onlinelibrary.wiley.com/doi/epdf/10.1111/phc3.12548>

42 ibid.

43 Ronald Dworkin, ‘Principle, Policy, Procedure’ in *A Matter of Principle* (Harvard University Press 1985). And see John Rawls, *A Theory of Justice* (OUP 1971).

44 ibid.

45 Dennis Galligan, *Due Process and Fair Procedures: A Study of Administrative Procedures* (Clarendon 1996) 101.

46 Jerry Mashaw, ‘Administrative Due Process: The Quest for a Dignitary Theory’ (1981) 61 Boston University Law Review 885.

47 Trevor Allen, ‘Procedural Fairness and the Duty of Respect’ (1998) 18 Oxford Journal of Legal Studies 497.

48 See Meyerson and MacKenzie (n 41) 5–6.

49 ibid, and see Denise Meyerson, Catriona Mackenzie and Therese MacDermott (eds), *Procedural Justice and Relational Theory: Empirical, Philosophical, and Legal Perspectives* (Routledge 2021).

50 See, for a fuller discussion in the context of law, Jennifer Nedelsky, *Law’s Relations* (OUP 2011).

51 Meyerson and MackKenzie (n 41).

52 ibid.

53 Catriona Mackenzie and Natalie Stoljar (eds), *Relational Autonomy: Feminist Perspectives on Autonomy, Agency and the Social Self* (OUP 2000).

54 Denise Meyerson, Catriona Mackenzie and Therese MacDermott (eds), ‘Introduction: Procedural Justice in Law, Psychology, and Philosophy’ in *Procedural Justice and Relational Theory: Empirical, Philosophical, and Legal Perspectives* (Routledge 2021) 4.

55 ibid.

56 ibid.

57 Kristina Murphy, ‘Procedural Justice and Its Role in Promoting Voluntary Compliance’ in P Drahos (ed), *Regulatory Theory: Foundations and Applications* (ANU Press 2017) 43; and see Tom Tyler, ‘Procedural Justice and the Courts’ (2017) 44 *Court Review: The Journal of the American Judges Association*) 26.

58 Meyerson, Mackenzie and MacDermott (n 49).

59 ibid 9.

60 Kirsten Rundle, ‘Administrative Discretion and Governing Relationships: Situating Procedural Fairness’ in Denise Meyerson, Catriona Mackenzie and Therese MacDermott (eds), *Procedural Justice and Relational Theory: Empirical, Philosophical, and Legal Perspectives* (Routledge 2021) 234.

61 ibid 235.

62 ibid.

63 ibid 236.

64 1990 Act, s 31ZA(1)–(4).

65 Donors who donated anonymously between 1 August 1991 and 31 March 2005 are (if they are aware the process exists) able to contact the HFEA and remove their anonymity, thus making themselves identifiable. As a result, for the cohort of donor-conceived people conceived of donations made during this period, there is a possibility that their donor’s information will be available.

66 Adoption and Children Act 2002.

67
*Rose* (n 24).

68 The HFEA is required by 1990 Act, s 31ZA(3) to give applicants a suitable opportunity to receive proper counselling.

69 We note, however, that by an Authority decision in January 2024, funding for support services will be withdrawn later this year and counselling replaced by web-based information and resources.

70
*Re H (A Minor) (Blood Tests: Parental Rights)* [1996] 3 FCR 201 at 220, per Lord Justice Ward.

71 Tomlinson (n 8) para 28.

72 Hurst (n 8) para 22.

73 Warnock Report (n 1).

74 In the UK, a significant minority of parents of young donor-conceived children do not intend to tell them about the circumstances of their conception. See Joanna Lysons and others, ‘Families Created via Identity-Release Egg Donation: Disclosure and an Exploration of Donor Threat in Early Childhood’ RBMO (2023) <doi.org/10.1016/j.rbmo.2023.05.007> accessed 29 January 2024.

75 Nixon (n 11) 3.

76 ibid 2.

77 ibid.

78 Barnwell (n 12) 1111.

79 ibid.

80 Warnock Report (n 1).

81
*Odièvre v France* (n 27), See Joint Dissenting opinion of Judges Wildhaber, Bratza, Bonello, Loucaides, Cabral Barreto, Tulkens, and Pellonpaa.

82 ibid paras [7–8].

83 World cafes are a face-to-face format for group dialogue underpinned by five principles, an informal setting, a warm welcome, three or more rounds of group discussion, with a table host, specific questions for discussion, and ‘harvesting’ insights via shared reflection and writing on large pieces of paper. The question here was ‘what is the role of law in online DNA testing?’.

84 HFEA (n 16) 15.

85 HFEA LRAG discussion document, ‘Donor Anonymity and Information Provision’ 7 <www.hfea.gov.uk/media/nacb35fx/lrag-discussion-paper-donor-anonymity-and-information-provision-2022-05-27.pdf> accessed 31 October 2023.

86 The Assisted Reproductive Treatment Amendment Act 2016 (Vic) (which came into force on 1 March 2017).

87 HFEA (n 16) Q22.

88 However, some of our participants have experience of the fact that DTCGT/social media searching for genetic ‘matches’ does not always allow identification of the genetic relations sought.

89 Fiona Darroch and Ian Smith, ‘Establishing Identity: How Direct-to-Consumer Genetic Testing Challenges the Assumption of Donor Anonymity’ Family Court Review (2021) <doi.org/10.1111/fcre.12553> accessed 30 January 2024.

90 See eg, ‘23andMe Terms of Service’ <www.23andme.com/en-gb/legal/terms-of-service> accessed 30 January 2024.

91 The Assisted Reproductive Treatment Amendment Act 2016 (Vic).

92 The Assisted Reproductive Treatment Amendment Act 2016 (Vic).

93 Department of Health & Human Services, ‘A Right to Know Your Identity’ (2015) 9 <www.health.vic.gov.au/sites/default/files/migrated/files/collections/policies-and-guidelines/a/a-right-to-know-your-identity.pdf> accessed 30 January 2024.

94 Note that the HFEA (n 39, para 27) acknowledges, by reference to the welfare of the child, the importance of such knowledge.

95 ibid 9.

96 HFEA, ‘Modernising Fertility Law’, s 2, proposal 8 <www.hfea.gov.uk/about-us/modernising-the-regulation-of-fertility-treatment-and-research-involving-human-embryos/modernising-fertility-law/#section-2> accessed 30 January 2024.

97 ibid.

98 Adoption Agencies Regulations 1976 (S.I., 1976, No 1796).

99 Hansard, 27 January 1977, Mr Phillip Whitehead (Derby North).

100 HFEA (n 103).

101 For the detail of the decision, see HFEA meeting documents for January and March 2024 <www.hfea.gov.uk/about-us/our-people/authority-meetings/> accessed 24 May 2024.

102 1990 Act s 31ZA(3)(b).

103 Meyerson, Mackenzie and MacDermott (n 49).

